# 2335 A mixed-methods evaluation to improve sustainability of community health coalition partnerships, activities, and impact on county-level health

**DOI:** 10.1017/cts.2018.273

**Published:** 2018-11-21

**Authors:** Jennifer Mansfield, Donna Vandergraff, Krystal Lynch, Douglas Miller, Dennis Savaiano

**Affiliations:** Indiana University School of Medicine

## Abstract

OBJECTIVES/SPECIFIC AIMS: Community health coalitions (CHC) aim to improve local cultures of health, health behaviors, and health outcomes. However, challenges sustaining partnerships and activities limit CHC impact. Traditional CHC evaluations survey members about perceived effectiveness, failing to capture underlying network structures and community health outcomes. Thus, we applied a mixed-methods evaluation in eight rural Indiana CHC, triangulating social network analysis [(SNA), conducted in 2017], functioning effectiveness [Coalition Self-Assessment Survey (CSAS), also 2017], and latest county health statistics (2015–2016) to assess existing CHC building efforts, inform best practices, and facilitate the adoption of evidence-based programming. METHODS/STUDY POPULATION: Across the eight rural Indiana CHC, relationships between the three evaluation components were analyzed using Pearson’s correlations. We are now collaborating with Purdue’s Nutrition Education Program Community Wellness Coordinators to scale up evaluation efforts throughout Indiana. RESULTS/ANTICIPATED RESULTS: CHC effectiveness was positively correlated with the average number of connections CHC members held in the network (mean indegree) and negatively correlated with the presence of a network broker (eigenvector centrality). However, effective leadership was positively correlated with opioid deaths and treatment, food insecurity, smoking during pregnancy, lack of healthcare coverage, and fair/ poor health status, and negatively correlated with prenatal care. Effective operating norms was positively correlated with smoking during pregnancy and preterm births, and negatively correlated with prenatal care. Effective action outcomes was positively correlated with opioid deaths and treatments, smoking during pregnancy, preterm births, and fair/ poor health status, and negatively correlated with respondents reporting they had no personal doctor. DISCUSSION/SIGNIFICANCE OF IMPACT: Interestingly, CHC effectiveness was positively correlated with poor county health outcomes related to infant well-being. Thus, CHC may develop in counties with a high unmet need for effective pregnancy and infant services. Alternatively, the prevalent CHC focus on obesity prevention may eclipse programmatic efforts to improve infant well-being. Longitudinal evaluations and scaling up evaluation efforts across Indiana are being pursued to clarify trajectories and inform best practices, which in turn should provide recommendations for network structures to improve CHC effectiveness and county health.

